# To Study the β-Globin Haplotype Pattern of Descent of a Set of Linked Alleles Occurring on the Same Chromosome in the Northern Province of India

**DOI:** 10.7759/cureus.36569

**Published:** 2023-03-23

**Authors:** Nitu Nigam, Swasti Sinha, Nishant Verma, Harish Gupta, Ghizal Fatima, Surbhi Gupta, Praveen Kumar

**Affiliations:** 1 Center for Advance Research, Cytogenetics Lab, King George's Medical University, Lucknow, IND; 2 Hematology and Oncology/Clinical Hematology, King George's Medical University, Lucknow, IND; 3 Pediatrics, King George's Medical University, Lucknow, IND; 4 Medicine, King George's Medical University, Lucknow, IND; 5 Public Health, Era's Lucknow Medical College and Hospital, Lucknow, IND; 6 Cytogenetics Lab, King George's Medical University, Lucknow, IND

**Keywords:** β-globin, beta thalassemia, restriction fragment length polymorphism, restriction endonuclease, rflp, north india, β-thalassemia major, haplotypes

## Abstract

Objective: To study the five mutations commonly prevalent in North India, i.e., IVS-I-5 (G→C), 619 bp deletion, IVS-I-1 (G→T), codon 41/42 (-TTCT), and codon 8/9 (+G), in the beta thalassemia (β-thalassemia) major children. The specific β-thalassemia mutations of different haplotype patterns of the β-globin gene cluster will also be determined.

Methods: A total of 125 children diagnosed with β-thalassemia major visiting the Department of Pediatrics of King George's Medical University were involved in the study. As per the QIAamp (Qiagen, Hilden, Germany) manufacturer guidelines, genomic DNA was isolated from whole blood. To identify the haplotype pattern within the β-globin gene cluster, the polymerase chain reaction-restriction fragment length polymorphism (PCR-RFLP) analysis was used. The respective restriction endonucleases used were *Hind III/GƔ, Hinc II/Ψß, Hinf I/ß, Ava II/ß, *and* BamHI* for the haplotype analysis in the β-globin pattern of descent of a set of linked alleles occurring on the same chromosome.

Results: Among the five common mutations, 73 patients had IVS-I-5 (G→C), 28 patients had 619 bp deletion, 17 patients had IVS-I-1 (G→T), five patients had Cd 41/42 (-TTCT), and two patients had Cd 8/9 (+G) mutations. Fifteen haplotypes (haplotypes 1-15) were identified in 125 β-thalassemia major children. Among the five haplotypes observed in the IVS-I-5 (G→C) mutation, the H1 haplotype was most predominant with a frequency of 27.2%, followed by the H2, H4, H3, and H10 haplotypes in the given population. In 619 bp deletion, IVS-I-1 (G→T), codon 41/42, and codon 8/9, haplotype H9, H12, H11, and H5 were seen, respectively.

Conclusion: β-thalassemia was found to be the most common in the northern province of Uttar Pradesh. The linkage of β-globin gene haplotypes with β-thalassemia mutations was explored in the northern province of Uttar Pradesh. The population of different natives is being mixed up due to migration and industrialization. These were some reasons for the occurrence of haplotypic heterogeneity. This haplotype heterogeneity was correlated with the origin of these mutations found to be unlike the origin of common ones from different provinces.

## Introduction

Beta thalassemia (β-thalassemia) is generally called Cooley's anemia. Thomas Cooley was the first scientist who discovered thalassemia. It is most frequent in the Middle East, Africa, and some Asian countries, including India [1]. Thalassemia is a single-gene disorder characterized by the absence or reduction of the β-globin chain. The β-globin gene is situated on chromosome 11 and leads to improper transcription due to mutations in the genes [1]. There are three types of thalassemia patients: thalassemia minor, thalassemia major, and thalassemia intermedia [2]. Among the three, thalassemia major patients have to depend on regular blood transfusion every three weeks, which leads to an overload of iron in several body organs. On the contrary, traits of thalassemia include a clinically healthy normal individual with microcytic anemia, elevated hemoglobin A2 (HbA2) levels, and mild hypochromic. The clinical variability of β-thalassemia major is based on inherited mutations among different populations. There are approximately more than 250 β-thalassemia mutations and 90% are caused by point mutations and 10% by different other types of mutations on the β-globin gene cluster [3,4].

Thalassemia is characterized as minor when one allele is impacted by mutation, intermedia when the disorder is mild, and thalassemia major when both alleles are impacted [5,6]. Thalassemia major patients are furthermore vulnerable to various complexities including infection of the liver, spleen, and gall bladder, and breakdown of RBCs that leads to hypoxia in the body. Severe anemia and reduced levels of oxygen result in low life expectancy in thalassemia major patients aged ~30 years. Because of these conditions, hepatomegaly, splenomegaly, growth retardation, jaundice, and dental issues are the major problems seen in β-thalassemia major patients.

Patients have to go through many tests to ensure investigation, including hematological and biochemical, serum transferrin, serum ferritin, complete blood count (CBC), peripheral blood smear, total iron-binding capacity (TIBC), urine urobilin, hemoglobin electrophoresis, and serum bilirubin [7]. After the hematological and biochemical tests, further molecular tests of the patients and their family members are done to analyze the types of mutation in that particular population. DNA assessment will be carried out for hereditary examination of the β-globin gene [8].

Worldwide, β-thalassemia arises due to various common, less common, and rare mutations because of insertions, deletions, and single nucleotide substitutions (SNPs), leading to frameshift mutations. Some of these reported mutations are Cd 15 (G→A), Cd 5 (-CT), Cd 30 (G→C), Cd 26 (G→T) (Hb-E), Cd 30 (G→A), Fr 8-9 (+G), Del 619 bp, Fr 47-48 (+ATCT), Fr 16 (-C), Fr 41-42 (-TTCT), IVSI-1 (G→T), IVSI-5 (G→C), IVSI-25 (25b del), IVSII-1 (G→A), and Cap +1 (A→C). These were categorized as βo with no formation of β chains and β+ with little β chain formation [9-13].

In β-thalassemia, numerous hereditary changes assume a critical part in determining the severity of the disease. Some studies show that a rise in fetal hemoglobin (HbF) level ameliorates the severity of β-thalassemia major, thus balancing the β- and α-globin chains. The presence of Xmn1 polymorphism, characterized by C→T transition at 158 bp upstream of the gamma-globin gene is responsible for increased HbF concentration, thereby reducing the thalassemic burden clinically as well as from the society [14,15]. It varies from population to population in the world.

The haplotype of the β-globin gene is helpful in determining genetic variations in a particular inhabitant dependent on the number and frequencies of haplotypes. Due to the migration of the inhabitants, there is a mixing up of the gene pool [16,17]. It has been found in some hemoglobinopathies such as sickle cell anemia comprising of the Arab-Indian (ARB) haplotype in Iran, the Indian subcontinent, and Eastern Arabian Peninsula; Benin (BEN) haplotype in Midwestern Africa; the Bantu haplotype in South Central and Eastern Africa [18,19]; Cameroon (CAM) haplotype along the west shore of Africa; and Senegal (SEN) haplotype in Atlantic West Africa. The aim of the study was to correlate the β-globin cluster haplotype pattern with human hereditary diversity to a particular β-thalassemia change.

In the current study, we enrolled β-thalassemia patients from the Pediatrics Department of King George's Medical University (KGMU) to determine their haplotype linkage. The main aim of the study was to perform a haplotype analysis of the β-globin gene to correlate the human genetic variation to a specific β-thalassemia mutation in the north Indian population. The second objective of this study was to link the observed haplotype pattern with the distinguishably found haplotypes worldwide.

## Materials and methods

Sample collection

The sample collection was done as per the Institutional Ethical Committee, KGMU, Lucknow, India (reference code: 89th ECM II B IMR-F/P2) prior to the commencement of the study. A total of 125 thalassemia major children were enrolled. The study was directed in accordance with the Declaration of Helsinki. The consent was attained from the participants prior to the blood collection from the β-thalassemia major patient visiting the Department of Pediatrics of KGMU.

DNA isolation

A total of 2 ml of venous blood was collected in ethylenediaminetetraacetic acid (EDTA) vials. Genomic DNA extraction was done from white blood cells in peripheral whole blood samples by using QIAamp (Qiagen, Hilden, Germany) as per the manufacturer's guidelines. The quantification of the isolated DNA was done by Nanodrop 1000 Spectrophotometer (Thermo Fisher Scientific, Waltham, MA).

**Figure 1:**
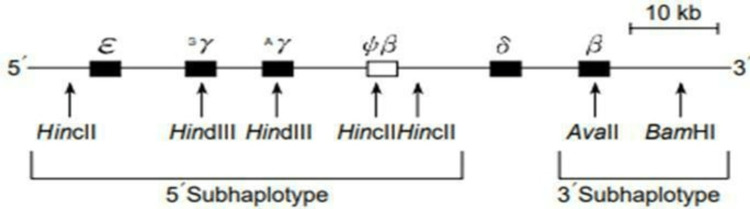
Seven RFLP sites in the β-globin gene cluster on chromosome 11. Black boxes indicate functional genes and the white box indicates a pseudogene. A relatively increased rate of recombination between δ- and β-globin genes has led to the subdivision of the full haplotype into 5′ and 3′ subhaplotypes. RFLP: restriction fragment length polymorphism.

The samples were screened by five restriction enzymes via restriction fragment length polymorphism (RFLP): *Hinc II*, *Hind III*, *Hinf I*, *Ava II*, and *BamHI* [20]. The β-globin gene cluster and the location of the different restriction sites used in the study are shown in Figure 1 [21]. The targeted restriction sites were amplified through polymerase chain reaction (PCR) using mutation-specific primers and procedures [22-24]. The appropriate enzymes were used to digest the amplicon followed by agarose gel electrophoresis.

Beta globin gene cluster haplotyping

The developmental patterns of β-globin gene expression are as follows. The β-globin consists of five expressed genes (ε, Aγ, Gγ, δ, and β) and one pseudogene (ψβ). Moreover, it has a 9.1 kb region recombination “hotspot” between the 5’ and 3’ β-globin gene cluster. The haplotypes are divided into 5’β and 3’β-haplotypes by the recombination hotspot [25]. This study will focus on the β-thalassemia major children with IVS-I-5 (G→C), 619 bp deletion, IVS-I-1 (G→T), Cd 41/42 (-TTCT), and Cd 8/9 (+G), together with the whole region of the β-globin gene cluster. The evolutionary relationships between human populations have been widely used to elucidate the 5’β-haplotype. The five restriction sites on the β-globin gene included in this study to construct the haplotype patterns are *Hinc II/Ψß*, *Hind III/Gγ*, *Hinf I/ß*, *Ava II/ß*, and *BamHI*. The five forward and reverse mutation-specific primer pairs were used for the PCR reaction, which includes the five restriction sites (Table 1). The PCR reactions were carried out in a 30 μl final volume comprising of ready-to-use emerald Takara reaction mix from Thermo Fisher Scientific, 0.2 μM of forward and reverse primers, and 100 ng of DNA templates. The PCR amplification was performed using a PCR thermal cycler (Biometra Gradient Thermocycler, Analytik Jena GmbH, Jena, Germany). The amplicons were subsequently digested with five restriction enzymes. A total of 15 μl of each amplified PCR product was digested using 10 units of FastDigest restriction enzymes in FastDigest Green buffers (Fermentas Life Sciences, Waltham, MA) and incubated at 37°C for 60-90 minutes. The digested fragment by the enzymes run on agarose gel electrophoresis at 50 volts/cm for 45-60 minutes. The RFLP marker sites were determined by the loss or gain of recognition sites onto the restriction enzyme. The PCR products digested by the restriction endonuclease enzymes constitute a positive (+), and the absence of a cut fragment was shown as a negative (−) [26-29].

**Table 1:** Primer sequences and type of nucleotide changes identified by RFLP. RFLP: restriction fragment length polymorphism; F: forward; R: reverse.

Restriction site	Primer sequence 5’----3’	Size (bp)	Fragments after enzyme cut	
			RFLP (-)	RFLP (+)
Hind III/Gγ	F: AGTGCTGCAAGAAGAACAACTACC	1202	970, 232	273, 697, 232
	R: CTCTGCATCATGGGCACTGAGCTC			
Hinc II/Ψß	F: TAAGCAAGATTATTTCTGGTCTCT	996	996	371, 625
	R: GTACTCATACTTTAAGTCCTAACT			
Ava II/ß	F: ACTCCCAGGAGCAGGGAGGGCAGG	963	963	772, 191
	R: TTCGTCTGT TTCCCATTCTAA ACT			
Hinf I/ß	F: GGAGGTTAAAGTTTTGCTATG	341	341	213, 128
	R: GGGCCTATGATAGGGTAAT			
BamHI	F: GCCCACATCACCAAG GCAAT	1520	1520	292, 1228
	R: GCTCTACGGATGTGTGAGAT			

## Results

A total of 125 thalassemia major children were included in the study. Among the five common mutations, 73 patients had IVS-I-5 (G→C), 28 patients had 619 bp deletion, 17 patients had IVS-I-1 (G→T), five patients had Cd 41/42 (-TTCT), and two patients had Cd 8/9 (+G) mutations (Table 2).

**Table 2:** Association of five common β-thalassemia mutations linked to different haplotypes.

ß-thalassemic mutation	No. of patients	%	Haplotype
IVS I-5 (G→C)	73	58.40	H1, H2, H3, H4, H10
IVS I-1 (G→T)	17	13.60	H8, H12, H15
Cd 41/42 (-TTCT)	5	4	H11, H14
619 bp deletion	28	22.40	H6, H7, H9, H13
Cd 8/9 (+G)	2	1.6	H5
Total	125		

This haplotype analysis study has demonstrated that the mutation IVS-I-5 (G→C) is majorly found in the population of the northern province of India (Lucknow) and Cd 8/9 is the least affecting. It suggests that the more frequent mutations exhibited linkage with four to five different haplotypes. It states that they might have migrated from other states or regions. Data analysis was performed for each haplotype, linking its relation with the β-globin gene mutations.

Haplotype 1 was the utmost dominant with a frequency of 27.2% (Table 3), followed by H2 (12.0%), H9 (11.2%), H4 and H12 (16%), H3 (6.4%), and H6, H10, and H15 (14.4%). Of the haplotypes, 12.8% were uncommon (H5, H7, H8, H11, H13, and H14). Although IVSI-5 (G→C) was present in haplotypes H1, H2, H3, H4, and H10, haplotype 1 showed the strongest linkage with IVS-I-5 (G→C) mutation. Another most frequent mutation, 619 bp deletion, was related to four uncommon haplotypes (H6, H7, H9, and H13), whereas IVS I-1 (G→T) and Cd 41/42 showed a strong association with H8, H12, H15, H11, and H14, respectively. The lesser frequent mutations (Cd 8/9) show linkage with one haplotype (H5). The distribution of different identified haplotypes and their relation with 15 mutant alleles of the β-globin gene is represented in Table 3.

**Table 3:** Hinc II, Hind III, Hinf I, Ava II, and BamHI are the five restriction enzymes used for the PCR-RFLP to study haplotype analysis. PCR-RFLP: polymerase chain reaction-restriction fragment length polymorphism; +: digested by restriction enzymes; -: undigested by restriction enzymes.

Enzymes/haplotypes	Hinc II	Hind III	Hinf I	Ava II	BamHI	Mutation-specific frequency	Overall frequency
H1	-	-	+	+	-	0.465	0.272
H2	-	-	+	-	-	0.205	0.12
H3	-	-	+	+	+	0.109	0.64
H4	+	-	+	+	-	0.136	0.08
H5	-	-	-	+	-	1.000	0.016
H6	+	+	+	+	-	0.214	0.048
H7	+	+	+	-	-	0.178	0.04
H8	+	-	+	-	+	0.058	0.008
H9	-	-	-	+	+	0.500	0.112
H10	+	+	+	+	-	0.082	0.048
H11	-	+	-	+	-	0.800	0.032
H12	+	+	-	-	+	0.588	0.08
H13	+	-	-	+	-	0.107	0.024
H14	-	+	+	+	-	0.200	0.008
H15	+	-	+	+	+	0.352	0.048

## Discussion

In the present study, mainly five categories of mutations in the β-globin gene were known among 125 subjects in the northern area of Uttar Pradesh. The most dominant mutation was IVS-I-5 (G→C) with a percentage of 58.4%. The high incidence of the mutation can be explained either by the presence of ancestral gene defects or by gene flow from other areas. The second prevalent mutation detected was 619 bp deletion with a frequency of 22.4%, followed by IVS-I-1 (G→T) with a frequency of 13.6%, Cd 41/42 (-TTCT) with 4%, and Cd 8/9 (+G) mutation with a frequency of 1.6%. We studied the association of five polymorphic restriction enzyme sites of β-globin gene cluster haplotypes with the gene mutations. These polymorphic sites result in 15 different haplotypes (H1-H15) in 125 β-thalassemia major children.

The link of a mutation to more than one haplotype is generally steady with the crossing over of 5′ into the β-globin group. Haplotype H1 has the most grounded association with IVSI-5 (G→C) mutation with a recurrence of 46.57%. This aids a more precise finding of the most common mutation in Lucknow, the northern region of Uttar Pradesh. The predominant haplotype (H1) was linked with IVSI-5 (G→C) among 15 unique haplotypes that can be understood by the founder effect phenomenon. The mutation 619 bp deletion of β-globin gene defect was related to H6, H7, H9, and H13 variants in that the linkage with haplotype H9 (50%) was the maximum. This haplotype linkage also represents the typical individuals indicating that the mutation originated on the existing chromosomal background [18]. IVS I-1 (G→T) has a great association with haplotype H12 with a frequency of 58.82. Although Cd 41/42 (-TTCT) mutation was associated mainly with two haplotypes H11 and H14, the highest linking was with H11 (80%). These findings enable the detection of frequent gene defects through haplotype analyses when mutation study is unsuccessful. Remarkably, Cd 8/9 (+G) mutation was in linkage disequilibrium (LD) only with one haplotype (H5). As a result, the detection of this haplotype confirms the presence of Cd 8/9 (+G) mutation with about 100% accuracy. This haplotypic heterogeneity recommends a multicentric source of these mutations. We may conclude the origin of these mutations may be different from the origin of common ones.

## Conclusions

A predominant haplotype pattern was seen in β-thalassemia major patients visiting the Pediatrics Department of KGMU, Lucknow, situated in the northern province of Uttar Pradesh. In this study, we reported the association of β-globin gene cluster haplotypes with a common mutation in this region of the state. Haplotype 1 pattern was seen in 27.2% of the study subjects, in accordance with a unicentric origin and an apparent single origin with low genetic diversity. During the study, β-thalassemia patients from the northern province possessed a similar haplotype background for common mutations. Thus, from this study, it is concluded that haplotype 1 is the commonest haplotype IVS-I-5 (G→C) mutation.
